# Weight and skeletal muscle loss with cabozantinib in metastatic renal cell carcinoma

**DOI:** 10.1002/jcsm.13021

**Published:** 2022-07-28

**Authors:** Emeline Colomba, Carolina Alves Costa Silva, Gwénaël Le Teuff, Jamie Elmawieh, Daniel Afonso, Axelle Benchimol‐Zouari, Annalisa Guida, Lisa Derosa, Ronan Flippot, Bruno Raynard, Bernard Escudier, François Bidault, Laurence Albiges

**Affiliations:** ^1^ Cancer Medicine Department, Gustave Roussy Paris‐Saclay University Villejuif France; ^2^ Institut National de la Santé Et de la Recherche Médicale (INSERM) U1015 Equipe Labellisée—Ligue Nationale contre le Cancer Villejuif France; ^3^ Biostatistics and Epidemiology Department Gustave Roussy Villejuif France; ^4^ Oncostat U1018, Inserm, Labeled Ligue Contre le Cancer University Paris‐Saclay Villejuif France; ^5^ Department of Anaesthesia, Surgery and Interventional, Gustave Roussy Paris‐Saclay University Villejuif France; ^6^ Imaging Department, Gustave Roussy Paris‐Saclay University Villejuif France; ^7^ Medical and Translational Oncology Unit, Department of Oncology Azienda Ospedaliera Santa Maria Terni Italy; ^8^ Dietetics and Nutrition Unit, Gustave Roussy Paris‐Saclay University Villejuif France

**Keywords:** Cabozantinib, Weight loss, Muscle wasting, Sarcopenia, Metastatic renal cell carcinoma

## Abstract

**Background:**

Cabozantinib, a standard of care metastatic renal cell carcinoma (mRCC), may be associated with weight and muscle loss. These effects of new generation VEGFR tyrosine kinase inhibitor on muscle mass loss are poorly described.

**Methods:**

All cabozantinib‐treated mRCC patients from January 2014 to February 2019 in our institution were included. Clinical data including weight were collected during therapy. Computed tomography images were centrally reviewed for response assessment, and axial sections at the third lumbar vertebrae were used to measure the total muscle area. Toxicities and cabozantinib outcomes were evaluated. Co‐primary endpoints included skeletal muscle loss and weight loss (WL), longitudinally evaluated during treatment. WL has been classified according to CTCAEv5.0: Grade 1 (loss of 5 to <10% of baseline body weight), Grade 2 (loss of 10% to <20% of baseline body weight), and Grades 3–4 (loss >20% of baseline body weight).

**Results:**

Patients were mostly men (70.3%), median age was 59.2 (range: 22.0–78.0) years, and median baseline body mass index was 25.0 (range: 16.4–49.3) kg/cm^2^. Prognosis according to International Metastatic RCC Database Consortium score was good, intermediate, and poor for 13 (13.0%), 63 (63.0%), and 24 (24.0%) patients, respectively. Out of a total of 120 patients, 101 patients with a median follow‐up of 22.3 months (range: 4.5–62.2) were eligible for analysis; 85 experienced muscle loss and muscle loss >10% increased during cabozantinib exposition, especially after 6 months of treatment. At cabozantinib baseline, 71 patients (70.3%) had sarcopenia, and 16/30 (53.3%) non‐sarcopenic patients developed sarcopenia during treatment. Baseline sarcopenia was associated with lower response rates (*P* = 0.031) and higher grades 3–4 toxicities (*P* = 0.001). Out of 92 patients included in the WL analysis, 44 (47.8%) and 12 (13.0%) experienced grades 2 and 3 WL, respectively.

**Conclusions:**

We report a high incidence of grades 3–4 WL, fourth times higher than reported in prior pivotal trials, and half of the patients developed sarcopenia while on cabozantinib treatment. Weight and muscle mass loss with cabozantinib are underreported and may require further investigations and early management.

## Introduction

Weight loss (WL) and sarcopenia are serious concerns in oncology.^1^, ^2^, ^3^ They have been associated with impaired quality of life, decreased overall survival (OS), and increased toxicities from oncologic treatments. More than one‐third of patients with cancer experience WL during the course of the disease,^4^ which leads to impaired physical performances and thus continuous deterioration of the patient's overall state and well‐being.^5^ Cancer‐associated cachexia (CAC) is a disorder characterized by loss of body weight with specific losses of skeletal muscle and adipose tissue that may lead to WL and sarcopenia.^6^, ^7^

Sarcopenia is a muscle disease rooted in adverse muscle changes that accrue across a lifetime.^8^ It is characterized by low skeletal muscle mass with impaired muscle function of multifactorial aetiology.^9^ It has been reported during vascular endothelial growth factor receptor (VEGFR) tyrosine kinase inhibitors (TKI) treatment.^10^ Several studies showed that sarcopenia is related to higher toxicity, poor responses to antineoplastic drugs, and decreased survival in cancer patients, including metastatic renal cell carcinoma (mRCC).^3^, ^11^, ^12^, ^13^, ^14^ Also, low body mass index and sarcopenia were associated to dose‐limiting toxicities of a VEGFR‐TKI in mRCC.^15^ In addition, the use of first generation VEGFR‐TKI induces around 1–2% of Grades 3–4 WL in prospective trials.^16^ Cabozantinib is a potent inhibitor of tyrosine kinases including VEGFR, c‐MET, and AXL4, currently approved for treatment of mRCC in first‐line treatment for intermediate and poor risk patients, and in second‐line treatment or beyond, after VEGFR‐targeted agents.^17^, ^18^ Cabozantinib is associated with high overall toxicity, with 68% of Grades 3–4 toxicity.^19^, ^20^, ^21^ Grade 3 WL is reported in 3% of patients under cabozantinib in pivotal trials,^19^, ^22^ and sarcopenia under cabozantinib has not been evaluated.

In the rapidly evolving environment of systemic therapy for mRCC,^23^, ^24^, ^25^ cabozantinib stands as a common second‐line treatment after current first‐line combinations with immune‐oncology (IO) therapy (IO‐IO or IO‐TKI) and has proven its efficacy in first‐line treatment in combination with nivolumab.^26^ The aims of this study are to (i) describe skeletal muscle loss ultimately leading to sarcopenia and WL during cabozantinib treatment and (ii) evaluate their impact on mRCC patients' outcomes.

## Patients and methods

### Patients

All consecutive patients treated with cabozantinib from January 2014 to February 2019 at Gustave Roussy for mRCC were included. Patients with early treatment discontinuation (less than 15 days) or missing data for baseline CT were excluded from analysis. For WL analysis, we excluded patients whose weight could be misinterpreted because of an oedematous syndrome (ascites or anasarca) appearing at least once before cabozantinib initiation or before its discontinuation. Clinical [age, gender, performance status, weight, body mass index (BMI), International Metastatic RCC Database Consortium (IMDC) score], laboratory, toxicity, and survival data were taken from the Gustave Roussy Institute Renal Cell Carcinoma (IGReCC) database. Main toxicity items included diarrhoea, nausea, decreased appetite, stomach pain, hand‐foot syndrome, hypothyroidism, or stomatitis.

### Anthropometry and body composition methods

Body weight was prospectively collected with a mechanical scale by the medical oncologist at the cabozantinib initiation (baseline) and every 2–6 weeks during the follow‐up visits, during the entire course of the treatment. WL is defined by a decrease of the total body weight compared with baseline. WL has been classified according to CTCAEv5.0: Grade 1 (loss of 5% to <10% of baseline body weight), Grade 2 (loss of 10% to <20% of baseline body weight), and Grades 3–4 (loss >20% of baseline body weight).

Skeletal muscle mass was measured using routine CT that was performed before cabozantinib initiation (baseline value) and then every 12 weeks (±4 weeks) during treatment for response assessment purposes. Two consecutive slices from the middle of the third lumbar vertebrae (L3) landmark were obtained for each patient.^27^ Image sections were analysed by a trained person (CACS and DA) using the SliceOmatic software V5.0 (Tomovision) and established thresholds of skeletal muscle tissue density (−29 to +150 Hounsfield units).^28^ Cross‐sectional areas of the sum of all L3‐containing muscles (psoas, paraspinal, and abdominal wall muscles) were measured in cm^2^ unit per slice, and the average value of both consecutive images was used as the total muscle area (TMA) for each patient. Skeletal muscle gain and loss were calculated as any variation of baseline TMA during treatment. TMA was normalized for stature to calculate skeletal muscle index, expressed in cm^2^/m^2^. Sarcopenia was defined as a skeletal muscle index lower than a pre‐specified sex‐based threshold: 55.4 for men and 38.9 for women.^29^

### Cabozantinib efficacy

A senior radiologist (ABZ) reviewed all CT scans to assess the response according to the Response Evaluation Criteria in Solid Tumor (RECIST) 1.1.

### Statistical analysis

Descriptive analyses were conducted to present the characteristics of mRCC patients treated by cabozantinib. Duration of cabozantinib exposure, defined as the time from baseline to discontinuation of treatment for any reason, was reported. Disease control rate (DCR), defined as complete response (CR) plus partial response (PR) plus stable disease (SD): (CR + PR + SD), and objective response rate (ORR), defined as CR + PR was calculated. Different time‐to‐event were described: (i) progression‐free survival (PFS), defined as the time from baseline to the date of progression or death from any cause, (ii) OS defined as the time from baseline to the date of the last follow‐up or death, (iii) dose‐modification‐free survival (DMFS) defined as the time from baseline to dose reduction (DR) and/or treatment discontinuation due to toxicity, and (iv) treatment‐failure‐free survival (TFFS) defined as the time from baseline to the date of progression or treatment discontinuation for toxicity. These time‐to‐events were estimated by the Kaplan–Meier method. Grades 3–4 adverse events (AE) were reported according to CTCAEv5.0. Specific analyses were performed for WL and skeletal muscle data each and both based on complete cases.

Co‐primary objectives were a description of skeletal muscle loss and WL during cabozantinib, defined as the smallest relative change from baseline. Skeletal muscle and WL change were described through the individual patient profiles, and the relative changes occurring from baseline to 1, 3, 6, 9, and 12 months. Time to first grade 2 WL (≥10%) and grade 3 WL (≥20%) from baseline was calculated.

Exploratory/secondary objectives included an evaluation of the following:

- WL in patients experiencing clinical benefit (CR + PR + SD) of cabozantinib. A subgroup analysis was performed in patients receiving cabozantinib for at least 6 months and with no progression during these first 6 months.
- Skeletal muscle loss leading to sarcopenia. We performed an analysis including non‐sarcopenic patients at baseline. We calculated sarcopenia‐free survival (SFS), defined as the time from baseline to the first occurrence of sarcopenia. This analysis was estimated by using the Kaplan–Meier method. A swimmer plot was depicted to characterize sarcopenia events during the disease course of these patients.
- WL and baseline sarcopenia impact on mRCC patients' toxicity and outcomes. DCR, ORR, PFS, OS, DMFS, TFFS, grade > 3 toxicity, and DR were compared according to their sarcopenic status at baseline. We also evaluated the association between an occurrence of a WL grade ≥ 2 and ≥3 over time on PFS and OS through a time‐dependent Cox model adjusted on age, performance status, and IMDC.
- Investigation of the intra‐individual correlation between weight and skeletal muscle was assessed at different timepoints (appendix)

Statistical analyses were performed using the SAS software 9.4 and the rmcorr R package for repeated measures correlation (https://cran.r‐project.org/web/packages/rmcorr/index.html). The cut‐off deadline for the analysis was August 2020. Institutional ethical committee approval was obtained from Gustave Roussy.

## Results

### Patient's characteristics

**Figure 1:**
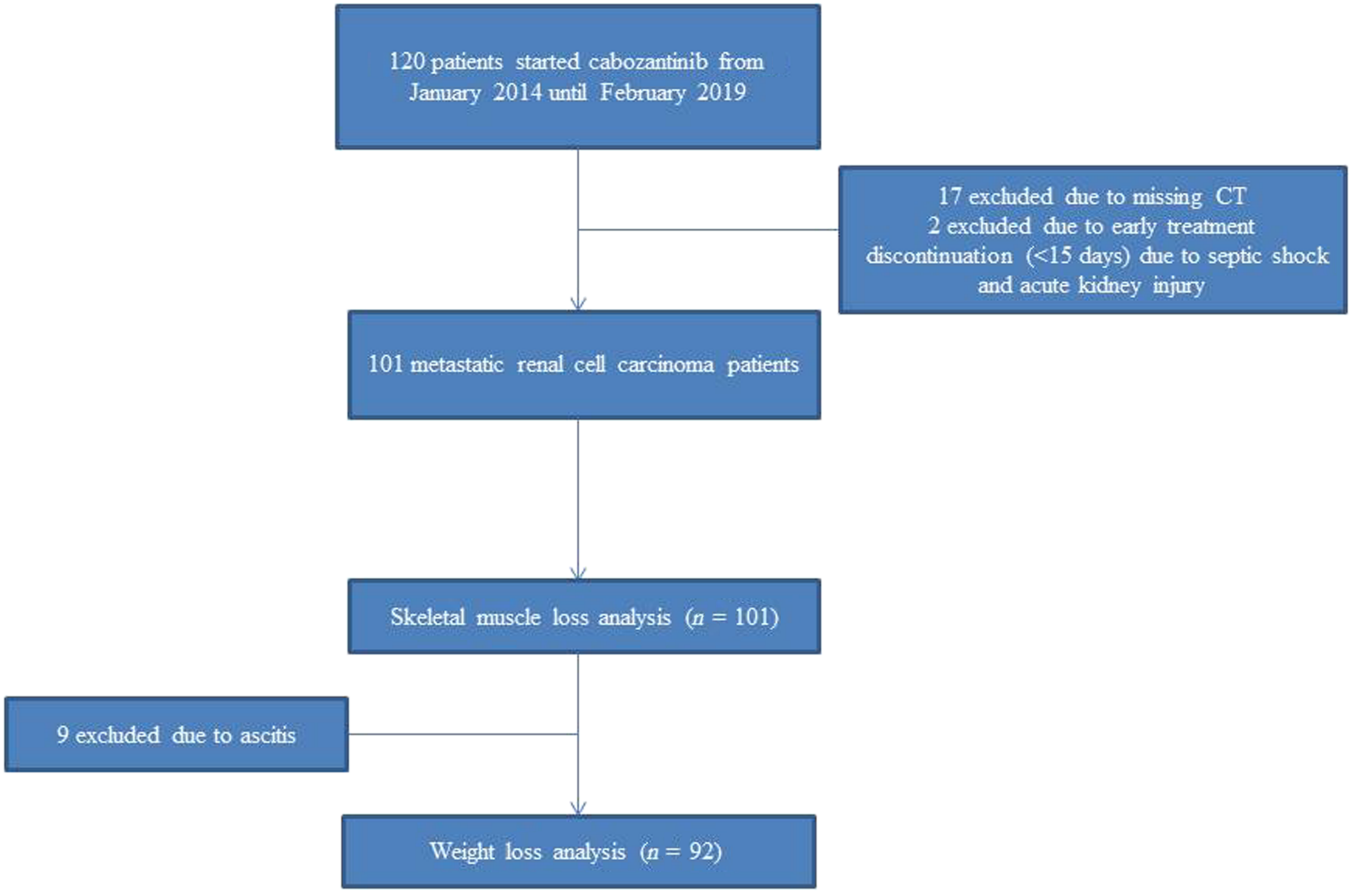
Patients flowchart.

Out of 120 patients with mRCC treated with cabozantinib from January 2014 to February 2019 at Gustave Roussy, 101 patients with a median follow‐up of 22.3 months (range: 4.5–62.2) were eligible for the current study (*Figure* 1). Seventeen patients were excluded due to missing CT and two patients due to early treatment discontinuation (one septic shock and one acute kidney failure).

Patients' characteristics and outcomes are reported in *Table* 1. Patients were mostly men (70.3%), median age was 59.2 (range: 22.0–78.0) years, and median baseline BMI was 25.0 (range: 16.4–49.3) kg/cm^2^. Prognosis according to IMDC was good, intermediate, and poor for 13 (13.0%), 63 (63.0%), and 24 (24.0%) patients, respectively (*Table* 1).

**Table 1:** Patients characteristics and outcomes according to weight (*n* = 92) and skeletal muscle loss (*n* = 101) set analysis

Characteristics		Skeletal muscle loss (*n* = 101), *n* (%)	Weight loss (*n* = 92), *n* (%)
Gender (male)		71 (70.3)	65 (70.7)
Prior nephrectomy		84 (83.2)	80 (87.0)
Weight (kg), mean [min–max]		74.0 [43.0–120.0]	74.1 [48.0–120.0]
Age (years), mean [min–max]		59.2 [22.0–78.0]	60.4 [28.0–78.0]
BMI (kg/cm^2^), mean [min–max]		25.0 [16.4–49.3]	25.0 [16.6–49.3]
BMI	<20	13 (13.1)	10 (10.9)
	[20–25]	43 (43.4)	41 (44.6)
	[25–30]	34 (34.3)	34 (37.0)
	≥30	9 (9.1)	7 (7.6)
	Missing, *n*	2	0
Histology	Clear cell RCC	75 (74.3)	72 (78.3)
	Non‐clear cell RCC	26 (25.7)	20 (21.7)
Performance status	0–1	73 (72.2)	68 (74.0)
	≥2	28 (27.7)	24 (26.1)
IMDC score at baseline			
Good		13 (13.0)	13 (14.3)
Intermediate		63 (63.0)	57 (62.6)
Poor		24 (24.0)	21 (23.1)
Missing, *n*		1	1
Previous systemic treatment			
0		3 (3.0)	3 (3.3)
1		31 (30.7)	27 (29.4)
2		27 (26.7)	26 (28.3)
≥3		40 (39.6)	36 (39.1)
Duration of exposure under cabozantinib (months), mean [min–max]		12.1 [1.0–32.1]	12.3 [1.0–32.1]
Sarcopenia at baseline			
Yes		30 (29.7)	—
No		71 (70.3)	—
Outcomes			
Disease control rate at 3 months		94 (93.1)	86 (93.5)
Objective response rate		37 (36.6)	35 (38.0)
Progression‐free survival (months), mean [min–max]		9.2 [7.6–10.6]	8.9 [7.4–10.1]
Overall survival (months), mean [min–max]		19.8 [17.7–23.7]	20.1 [17.7–29.7]
Overall Grades 3–4 toxicities (CTCAEv4)		35 (34.7)	33 (35.9)

BMI, body mass index; IMDC, International Metastatic RCC Database Consortium; SMA, skeletal muscle loss analysis.

Median time on cabozantinib was 12.1 months (range: 1.0–32.1). At the cut‐off date of the analysis, 31 patients (30.7%) were still on cabozantinib, while 52 (51.5%) had stopped for disease progression, 16 (15.8%) for serious toxicity (grades 3–4), and 2 (2.0%) for drug holidays. DR due to toxicity was required for 79 patients (78.2%).

DCR and ORR were 93.1% and 36.6%, respectively. Median PFS (98 events) and OS (54 deaths) were 9.2 months [7.6–10.6] and 19.8 months [17.5–23.7], respectively. Median DMFS (79 events) and TFFS (68 events) were 2.5 months [1.9–3.2] and 13.9 months [10.2–15.7], respectively. Twenty‐eight patients (36.8%) experienced Grades 3–4 AE.

### Weight loss analysis

**Figure 2:**
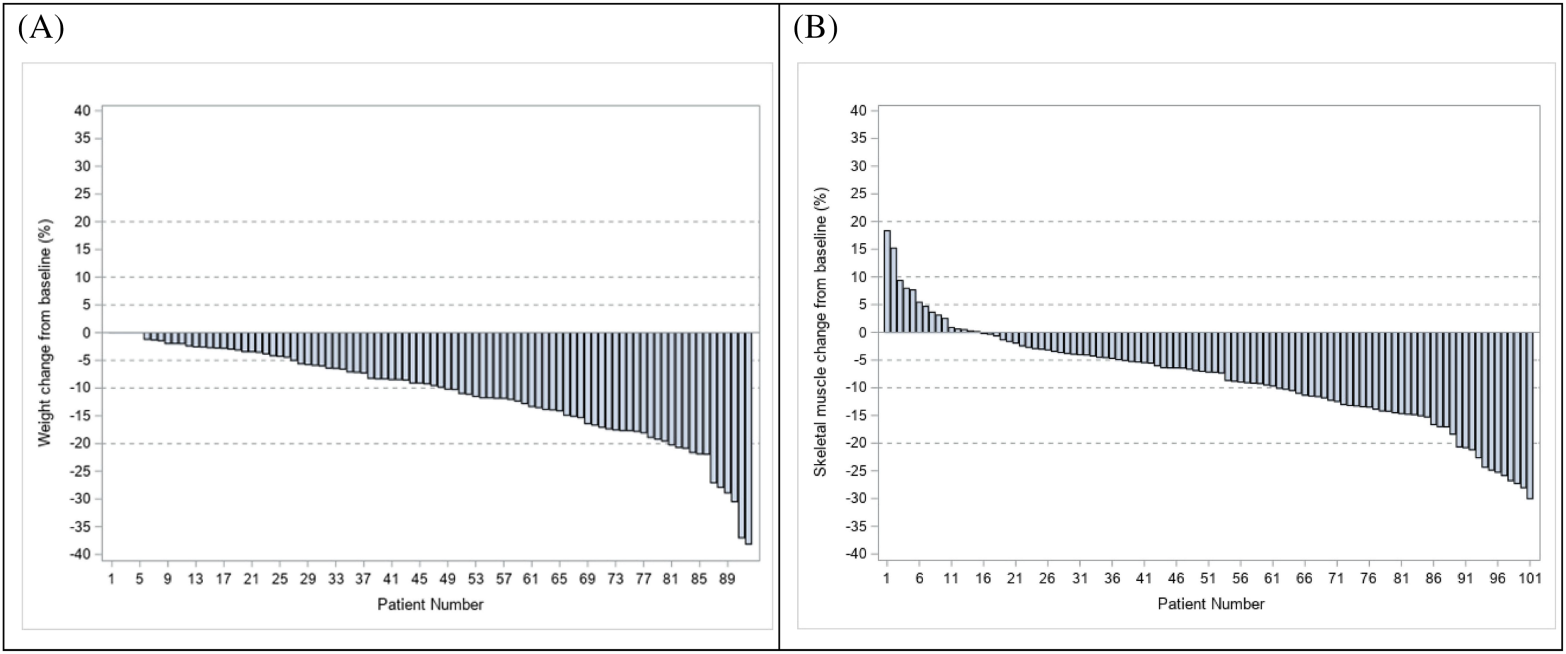
Maximal relative change of weight loss (*n* = 92) (*A*) and skeletal muscle (*n* = 101) (*B*) from baseline during cabozantinib treatment.

Ninety‐two patients were included in WL analysis with a median follow‐up of 22.6 months (range: 6.3–62.2). Patients' characteristics are presented in *Table* 1. Median time on cabozantinib was 12.3 months (range: 1.0–32.1). Twenty‐nine patients (31.5%) were still on cabozantinib, 47 (51.0%) stopped for progression, 13 (14.1%) for serious (grades 3–4) toxicity, and 3 (0.03%) for drug holiday. DCR and ORR were 93.5 [95%CI 88.8–98.5] and 38.0% [28.1–47.9], respectively. Median PFS (92 events) and OS (47 deaths) were 8.9 [7.3–10.1] and 20.1 months [17.7–29.7], respectively. Median DMFS (72 events) and TFFS (60 events) were 2.5 months [1.8–3.2] and 13.8 months [9.7–17.7], respectively. Thirty‐eight patients (41.4%) experienced grades 1–2 diarrhea and 28 patients (38.4%) had grades 3–4 AE. *Figure* S1A reports weight change during cabozantinib treatment for each patient. The WL occurred despite interventions such as DR in 70 (76.9%) patients, and nutritional support in 26 (28.2%) patients. *Figure* 2A depicts the waterfall plot of the relative change of body weight from baseline to the worst WL value. The median relative variation of body weight over time was −9.4% with the following distribution: grade 1 WL in 21 (22.8%), grade 2 WL in 32 (34.8%), and grade 3 WL in 12 patients (13.0%). The occurrence of grades 2 and 3–4 WL increased especially after 6 months of cabozantinib exposure. In patients under cabozantinib more than 6 months (median duration of 15.2 months, range: 6.5–32.1) with no progression within the first 6 months (*n* = 64, 69.6%), the incidence of WL was higher than in the overall population: 35 patients (54.7%) had a grade 2 WL with a median cumulative probability of 12.0 months [9.2–21.1] and 9 patients (14.1%) had grades 3–4 WL with a median cumulative probability not reached (refer to *Figures* 2A and S1A for maximal relative change and spider plot of change from baseline, respectively).

### Outcomes according to weight loss

The occurrence of a grade 2 WL considered as a time‐dependent variable and controlled for age (<, ≥70 years), performance status (0–1, ≥2), and IMDC was not associated to PFS [hazard ratio (HR) = 0.844 [0.513–1.389], *P* = 0.5048] and OS (HR = 0.779 [0.397–1.531], *P* = 0.4693). Similar results were observed for grades 3–4 WL (HR = 1.980 [0.861–4.555], *P* = 0.1079 and HR = 1.653 [0.673–4.065], *P* = 0.2732 for PFS and OS, respectively).

### Skeletal muscle loss analysis

Overall, 86 (85.2%) patients experienced skeletal muscle loss during the cabozantinib treatment. *Figure* S1B reports the overall patient muscle change during the cabozantinib treatment. *Figure* 2B reports skeletal muscle change from baseline to the worst value of skeletal muscle mass. The median relative variation of skeletal muscle over time is −8.3%. The distribution of greater muscle loss during the cabozantinib treatment is as follows: <5.0% in 22 patients (21.8%), 5‐10% in 24 patients (23.8%), 10‐20% in 28 patients (27.7%), and >20% in 12 patients (11.9%). Out of the 101 patients, 71 (70.3%) patients were sarcopenic at baseline while 30 patients (29.7%) were not. Patients' characteristics according to baseline sarcopenia are represented in *Table* S1. Sarcopenic patients were mostly males (80.3%) and had lower median baseline weight and BMI than non‐sarcopenic patients. Sarcopenic patients were mostly considered as underweight/normal weight (71.5%) according to BMI classification, while non‐sarcopenic patients were overweight and obese (79.3%).

### Outcomes according to baseline sarcopenia

**Figure 3:**
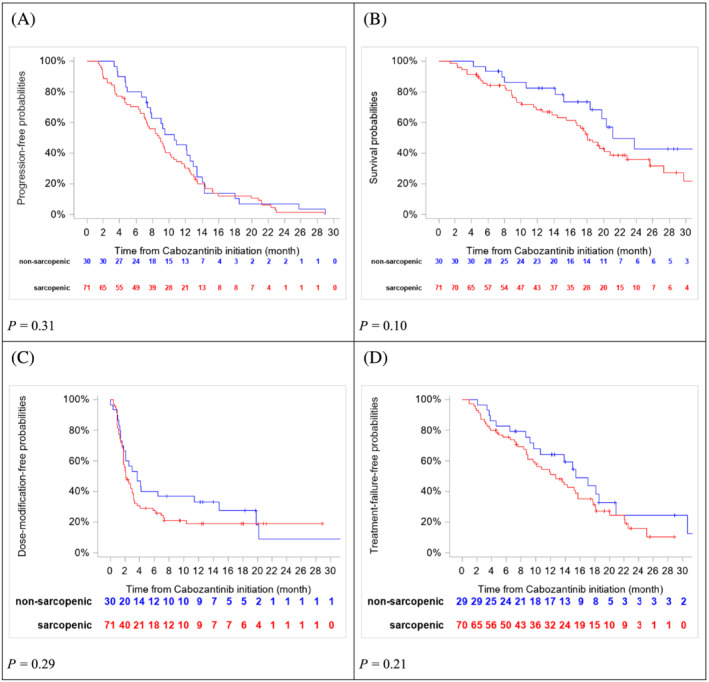
Progression‐free survival (*A*), overall survival (*B)*, dose‐modification‐free survival (*C*), and treatment‐failure‐free survival (*D*) according to sarcopenia at baseline.

Non‐sarcopenic and sarcopenic patients had similar DCR (100.0 vs. 90.1%; *P* = 0.10). Non‐sarcopenic patients had higher response rates to treatment than sarcopenic patients (53.3% vs. 29.6%; *P* = 0.024). The median PFS was 10.6 months [95% confidence interval (CI), 7.8–12.9; 29 events] and 8.9 months (95% CI, 7.1–10.1, 69 events) in non‐sarcopenic and sarcopenic patients, respectively (*P* = 0.31) (*Figure* 3A). The median OS was 21.1 months (95% CI, 18.3 to 62.2) and 18.1 months (95% CI, 15.5–22.8) for non‐sarcopenic and sarcopenic patients, respectively (*P* = 0.10) (*Figure* 3B). The median DMFS was 3.7 months (95% CI, 1.8–11.4) and 2.1 months (95% CI, 1.8–3.1) for non‐sarcopenic and sarcopenic patients, respectively (*P* = 0.29) (*Figure* 3C). Median TFFS was 15.4 months (95% CI, 9.7–20.9) and 12.7 months (95% CI, 8.9–15.7), for non‐sarcopenic (18 events) and sarcopenic (50 events) patients, respectively (*P* = 0.21) (*Figure* 3D). The percentage of grades 3–4 AE was significantly lower in non‐sarcopenic (10.0%, *n* = 3/30) than in sarcopenic patients (45.1%, 32/71) (*P* = 0.0007). DR was similar for sarcopenic (76.7%, 23/30) and non‐sarcopenic (75.7%, 53/70) patients (*P* = 0.92).

### Sarcopenia subgroup analysis in non‐sarcopenic patients at cabozantinib baseline

**Figure 4:**
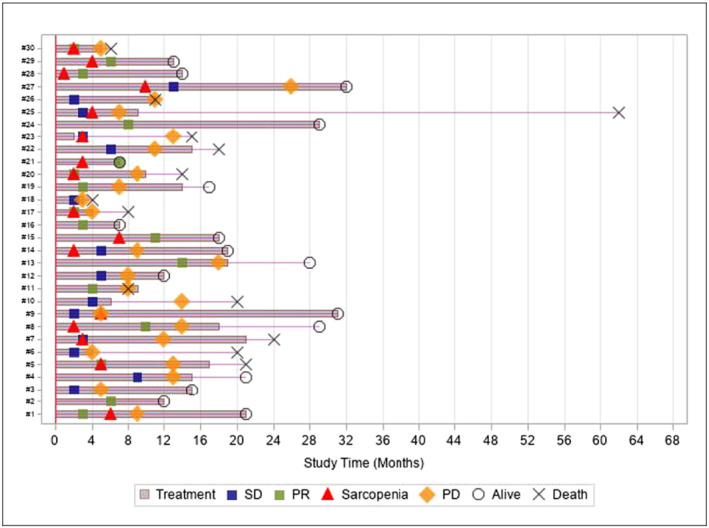
Swimmer plot of non‐sarcopenic patients at cabozantinib initiation (*n* = 30). PD, progression disease; PR, partial response; SD, stable disease.

*Figure* 4 describes the disease course for the 30 non‐sarcopenic patients. Out of 30 patients, 16 patients (53.3%) experienced sarcopenia over treatment exposure, with a median SFS of 6.4 months (95% CI, 4.3‐NR). And out of these, four patients experienced sarcopenia despite PR under cabozantinib. We reported in *Figure* 4 that half of patients that were not sarcopenic at baseline became sarcopenic during cabozantinib curse. It is not possible according to our result to differentiate whether the sarcopenia event was related to tumour progression or a cabozantinib effect comparing its appearance to patients' response status. Moreover, most of the patients did experience sarcopenia (10/15 patients) very earlier before the progression event. Sarcopenia and progression event seems relatively independent according to *Figure* 4.

### Investigating the association between weight and muscle skeletal

**Figure 5:**
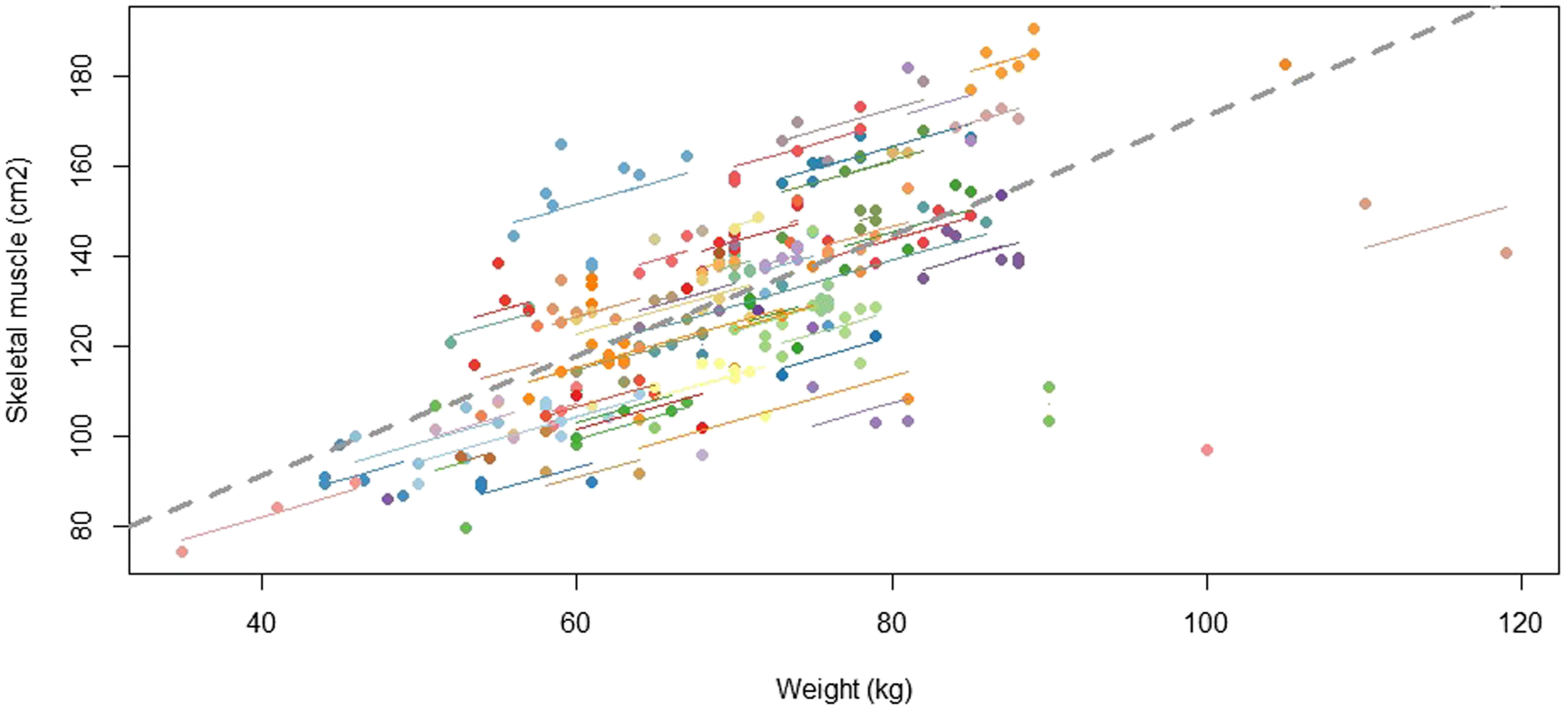
Scatter plot of the weight and skeletal muscle. Each dot with the same colour represents paired data (weight & muscle skeletal) for a patient with the corresponding line showing the repeated measure correlation fit for each patient.

*Figure* 5 depicts the scatterplot of the repeated measures paired data from 92 patients and 263 paired measures. Paired data from the same patient are given by the same colour with corresponding lines to show the intra‐patient linear association. The repeated measures correlation between weight and skeletal muscle is 0.53 [0.42–0.63] (*P* < 0.001) indicating that patients with higher body weight tend to have higher skeletal muscle mass. The sensitivity analysis using the time of body weight measures as the reference leads to repeated measures correlation of 0.42 [0.33–0.51] (*P* < 0.001) with 92 patients and 380 paired measures. In this sensitivity analysis, one measure of skeletal muscle can be paired to multiple body weight measures for a patient because the number of weight measures is higher than the number of skeletal muscle measures. Similar results were obtained after the standardization of body weight and skeletal muscle for height. In 92 overlapping patients between weight and skeletal muscle analysis set, out of 44 patients who had grade 2 WL, 36 were sarcopenic prior to the grade 2 WL. Out of 12 patients who had grade 3 WL, 10 were sarcopenic prior to the grade 3 WL. However, we also observed that sarcopenia appeared even though no grade WL occurred during cabozantinib treatment in 40 patients (43.5%).

## Discussion

We report the first study evaluating skeletal muscle loss leading to sarcopenia and WL during cabozantinib treatment in mRCC patients. To avoid a selection bias in this retrospective cohort, no specific inclusion/exclusion was used, and all consecutive patients treated with cabozantinib were included. We excluded patients who did not have data that were crucial for the study (weight measures or CT).

We reported higher DR (77%), compared with pivotal trials (60% in METEOR trial and 46% in CABOSUN).^19^, ^20^, ^22^ Even though, the incidence of grades 3–4 WL (13%) was also higher (*Table* 2).^19^, ^20^, ^22^ Our findings are consistent with previous data reported in advanced thyroid cancer treated with cabozantinib.^30^ In this study, 57.1% of patients had grade 2 WL after 3 months of therapy, and 21% had a grade 3 WL after 12 months of therapy. DR and therapy discontinuation were similar to our study, occurring in 71% and 43% of patients, respectively, and gastrointestinal toxicities were moderate. We found that the occurrence of grade ≥ 2 WL over time were not associated with survival, reinforcing the idea that WL is dependent on the cabozantinib dose and time of exposure.

**Table 2:** Summary of weight loss (WL) and gastrointestinal toxicities of antiangiogenics in prospective trials

Trial	Patients, *N*	Drug and schedule	Any grade WL, *N* (%)	G3–4 WL, *N* (%)	Any grade diarrhoea, *N* (%)	G3–4 diarrhoea, *N* (%)	Any grade stomatitis, *N* (%)	G3–4 stomatitis, *N* (%)	Any grade nausea, *N* (%)	G3–4 nausea, *N* (%)	Any grade anorexia, *N* (%)	G3–4 anorexia, *N* (%)	Any grade vomiting, *N* (%)	G3–4 vomiting, *N* (%)	Reference
METEOR	330	Cabozantinib 60 mg/day continuous	114 (34)	9 (3)	249 (75)	43 (13)	73 (22)	8 (2)	173 (52)	15 (5)	156 (47)	8 (2)	113 (34)	7 (2)	Choueri NEJM, 2015
CABOSUN	78	Cabozantinib 60 mg/day continuous	25 (32)	3 (4)	56 (72)	8 (10)	28 (36)	4 (5)	25 (32)	2 (3)	37 (47)	4 (5)	NR	NR	Choueri J Clin Oncol., 2017
	72	Sunitinib 50 mg/day 50 mg/g 4 weeks on/2 weeks off	12 (17)	0 (0)	38 (53)	8 (11)	21 (29)	4 (6)	28 (39)	3 (4)	23 (32)	0 (0)	NR	NR	
Axis	359	Axitinib 10 to 20 mg/day continuous	89 (25)	8 (2)	197 (55)	38 (11)	54 (15)	5 (1)	88 (27)	1 (<1)	105 (33)	3 (<1)	105 (33)	3 (<1)	Rini Lancet, 2011
	355	Sorafenib 600 mg/day continuous	74 (21)	5 (1)	189 (53)	26 (7)	44 (12)	1 (<1)	77 (22)	4 (1)	101 (29)	13 (4)	61 (17)	3 (1)	
Sunitinib vs. interferon‐alfa	355	Sunitinib 50 mg/g 4 weeks on 2 weeks off	NR	NR	53 (15)	5 (1)	25 (7)	1 (0.3)	44 (12)	3 (0.8)	NR	NR	24 (7)	4 (1)	Motzer NEJM, 2007
Comparz	548	Sunitinib 50 mg/g 4 weeks on/2 weeks off	33 (6)	1 (<1)	NR	NR	150 (27)	8 (1)	NR	NR	NR	NR	NR	NR	Motzer NEJM, 2013
	554	Pazopanib 800 mg/day continuous	84 (15)	5 (1)	NR	NR	77 (14)	4 (1)	NR	NR	NR	NR	NR	NR	
VEG105192	290	Pazopanib 800 mg/day continuous	NR	NR	150 (52)	11 (4)	NR	NR	74 (26)	2 (1)	65 (22)	6 (2)	61	7	Stenberg J Clin Oncol, 2010

G3–4, rate of Grades 3–4 adverse event according to CTCAEv5.0; NR, not reported.

Considering other prognostic factors that may influence WL (*Table* S2), median age was 63.0 years in METEOR and CABOSUN trials vs. 59.2 years in our real‐life cohort. We know that elderly patients are more prone to develop sarcopenia and WL. Although in our population the grades 3–4 WL is up to four times higher than the previous studies, our population was slightly younger. In other hand, our cohort was enriched in patients with poor prognosis according to IMDC score (twice more than in METEOR trial that investigated cabozantinib in the same setting after at least one line of VEGFR inhibitors). Indeed, patients were respectively good, intermediate, and poor according to IMDC score in 14.3%, 62.6%, and 23.1% in our study vs. 45%, 42%, and 12% in METEOR trial. In CABOSUN trial, the part of patients with poor prognosis was consistent with our population, but all patients were in first‐line systemic therapy setting. Patients from CABOSUN were not previously exposed to VEGR inhibitors that could explain the few WL incidence. In METEOR trial, only 29% of patients received at least two previous VEGFR inhibitors therapies. Whereas in our real‐life cohort, 66% received at least two line of systemic therapy indicating a population more heavily pretreated. Thus, a heavily pretreated population and more patients with poor prognosis IMDC score may explain the higher rate of grades 3–4 WL.

Few studies have longitudinally evaluated skeletal muscle during TKI treatment in mRCC patients. Antoun *et al*. reported that sorafenib‐treated mRCC patients (*N* = 80) progressively lost muscle mass during treatment and that the number of patients meeting the criteria for sarcopenia increased from 52.5% at sorafenib baseline to 71.0% after 1 year of treatment in a VEGFR‐TKI naïve population.^10^ Other studies in metastatic settings also reported a high sarcopenia prevalence from 33% to 90.3% before starting treatment with VEGFR‐TKI or mTOR inhibitors in mRCC.^12^, ^31^ In our study, the high prevalence of baseline sarcopenia (70%) can be attributable to our population of heavily pretreated patients, with 39.6% treated beyond fourth line. Furthermore, most patients (85.2%) experienced skeletal muscle loss, showing that even sarcopenic patients at baseline worsened their skeletal muscle mass during therapy. Skeletal muscle loss led to the development of sarcopenia in half of the patients that were non‐sarcopenic at baseline.

We reported in *Figure* 4 that half of patients (*n* = 16) that were not sarcopenic at baseline (*n* = 30) developed sarcopenia during cabozantinib course. From this swimmer plot that only reported different events over time in each patient, it is not possible to differentiate whether the development of sarcopenia was related to a cabozantinib effect, a tumour progression, or both. We observed that some patients who developed sarcopenia (i) did not have achieved best response (only two patients had achieved SD or PR as best response) and (ii) seven progressed after at least 4 months from sarcopenia event. Thus, sarcopenia and progression event seem relatively independent. We reported that sarcopenia is an early event during treatment, with a SFS shorter than PFS as reported in *Figure* 3. However, our descriptive study was not designed to demonstrate the causality link between cabozantinib and the development of sarcopenia.

Few retrospective studies in mRCC, reported sarcopenia during VEGFR‐TKI and mTOR treatment as an independent predictor of reduced OS.^12^, ^32^ In this study, PFS and OS were not significantly associated with sarcopenia at baseline, probably due to a lack of power calculation because of the low number of patients included in this study or to insufficient follow‐up. Concerning the toxicity, it is well known that body composition influences treatment‐related toxicity and outcomes by affecting tissue distribution and pharmacokinetics.^33^ Others studies in mRCC demonstrated higher incidence of dose‐limiting toxicity during sunitinib or sorafenib treatments in sarcopenic patients.^10^, ^34^, ^35^ Indeed, we reported that sarcopenia at baseline was associated with a significant higher grades 3–4 overall toxicity.

Our exploratory analyses investigating the intra‐individual correlation between weight and skeletal muscle demonstrated that most patients that experienced grades 2 or 3 WL were already sarcopenic before the WL event. It is known that WL and skeletal muscle loss can be affected to CAC. CAC is a disorder characterized by loss of body weight with specific losses of skeletal muscle and adipose tissue driven by a variable combination of reduced food intake and metabolic changes. It includes elevated energy expenditure, excess catabolism and inflammation.^6^, ^7^ At the tissue level, mechanisms include activation of inflammation, proteolysis, autophagy, and lipolysis. Our study is descriptive and was not able to determinate the impact of CAC in the WL and sarcopenia under cabozantinib but CAC may participate to the phenomenon in some patients. Our hypothesis is that WL is not only explained by usual gastrointestinal toxicities (diarrhea, stomach pain, anorexia, nausea, vomiting, and stomatitis) but also linked to a particular mechanism of the drug (*Figure* S2).

Probably, cabozantinib has a direct deleterious side effect on the muscle mass. Studies suggested an independent mechanism that leads to early‐onset skeletal muscle and adipose tissue loss. There is evidence that VEGFR‐TKI reduce skeletal muscle maturation and growth by molecular pathways.^11^, ^36^, ^37^ They are associated with the downstreaming of PI3K, AKT, and mTOR pathways that are implicated as key mediators in activating muscle protein synthesis by amino acids and other stimuli.^38^ Cabozantinib, as a multitargeted TKI and c‐MET inhibitor, may promote skeletal muscle loss by down‐regulating kinases involved in the regulation of muscle anabolism. During embryogenesis and in adult life, motility function of c‐MET is crucial for the long‐range migration of skeletal muscle progenitor cells.^39^ Cabozantinib may have an indirect effect as well. Indeed, some pro‐inflammatory cytokines are implicated in cachexia, such as interleukin‐1, interleukin‐6, and tumour necrosis factor‐alpha. Preclinical evidence show that cabozantinib exerts an effect in immune activation and cytokine release,^32^, ^40^ which could be implicated in WL and body composition changes during treatment.

Our data highlight the importance of nutritional assessment at cabozantinib baseline and close follow‐up during treatment. Usual gastrointestinal toxicity should be treated as recommended, and nutritional advice to increase energy and protein intake should include oral nutritional supplements and splitting meals.^5^ It is also known that inactivity causes muscle wasting, potentiates catabolic signals, and desensitizes muscle to anabolic factors.^41^, ^42^ So patients should be encouraged to practice adapted regular physical activity. Including physical activity in daily life, resistance and aerobic exercise training would help patients to promote their protein anabolism.^43^ This prevention may significantly reduce the toxicity of this powerful therapy.

## Conclusion

We report grades 3–4 WL four times higher than the incidence from registration trials and a high incidence of skeletal muscle loss leading to sarcopenia during cabozantinib treatment. Furthermore, sarcopenia was associated to a higher risk of grades 3–4 toxicity. These side effects are underevaluated in clinical trials and require more investigations and early management. In this context, early nutrition interventions are mandated. Ultimately, nutritional intervention to prevent patients from WL and sarcopenia could affect treatment outcomes and quality of life. The clinician's challenge is to maintain adequate dose intensity and quality of life in the meantime, to optimize the benefit of cabozantinib.

### List of where and when the study has been presented in part elsewhere

1. Silva, C.A.C.; Afonso, D.; Colomba, E.; Le Teuff, G.; Derosa, L.; Raynard, B.; Guida, A.; Benchimol‐Zouari, A.; Escudier, B.; Bidault, F; Albiges, L. Skeletal muscle loss as an adverse event during Cabozantinib treatment in patients with metastatic renal cell carcinoma. Annals of Oncology (2019) 30 (suppl_5): v356‐v402. 10.1093/annonc/mdz249
2. Colomba, E.C.; Silva, C.A.C.; Le Teuff, G.; Guida, A.; Raynard, B.; Derosa, L.; Benchimol‐Zouari, A; Escudier, B.; Albiges, L. Weight loss is an underestimated adverse event with cabozantinib in patients with metastastic renal cell carcinoma (mRCC). Annals of Oncology (2019) 30 (suppl_5): v356‐v402. 10.1093/annonc/mdz249

## Conflict of interest

E.C. declared consulting or Advisory Role: Ipsen, Sanofi, GSK, Merck, BMS and Pfizer. R.F. declared consulting BMS, Ipsen. B.R. received honoraria as a lecturer from Baxter, Fresenius Kabi, Nestle Health Science, Nutricia, and Amgen. B.E. declared consulting or Advisory Role: Bristol‐Myers Squibb, Ipsen, Roche, Pfizer, Oncorena, Aveo. L.A. declared consulting or Advisory Role: Novartis, Amgen (Inst), Bristol‐Myers Squibb, Bristol‐Myers Squibb (Inst), Ipsen (Inst), Roche (Inst), Novartis (Inst), Pfizer (Inst), Astellas Pharma (Inst), Merck (Inst). A.B.Z., F.B., C.A.C.S., L.D., G.L.T., E.J., and D.A. declared no disclosures.

## Supporting information


**Table S1:** Patients'characteristics and outcomes according to sarcopenia status at cabozantininb baseline (*n* = 101)
**Table S2**: Description of age and IMDC score criteria in METEOR, CABOSUN and current study
**Figure S1:** Spider splot of change from baseline of weight (A) and skeletal muscle (B) during cabozantinib treatment (patient level)
**Figure S2:** Hypothesis of mechanism of WL during cabozantinib
