# Supplementary material for: Weight and skeletal muscle loss with cabozantinib in metastatic renal cell carcinoma
Source: J Cachexia Sarcopenia Muscle. 2022 Jul 28;13(5):2405–16. doi: 10.1002/jcsm.13021 (PMC9530538; doi:10.1002/jcsm.13021)

**Supplementary materials:** 2 tables and 2 figures

**Supplementary Table 1**: Patients’characteristics and outcomes according to sarcopenia status at cabozantininb baseline (n=101)

| Characteristics | Non sarcopenic  (n=30)  n (%) | Sarcopenic  (n=71)  n (%) | Overall  (n=101)  n (%) |
| --- | --- | --- | --- |
| Gender Male | 14 (46.7) | 57 (80.3) | 71 (70.3) |
| Prior Nephrectomy | 26 (86.7) | 58 (81.7) | 84 (83.2) |
| Weight (mean) [min-max] kg | 80.4 [50.0-120.0] | 71.4 [43.0-101.0] | 74.0 [43.0-120.0] |
| Age (mean) [min-max] years | 60.8 [43.0-77.0] | 58.5 [22.0-78.0] | 59.2 [22.0-78.0] |
| BMI (mean) [min-max]kg/cm2 | 28.7 [20.7-49.3] | 23.5 [16.4-35.8] | 25.0 [16.4-49.3] |
| BMI <20  [20-25[  [25-30[  ≥30  Missing | 0 (0.0)  6 (20.7)  16 (55.2)  7 (24.1)  1 | 13 (18.6)  37 (52.9)  18 (25.7)  2 (2.9)  1 | 13 (13.1)  43 (43.4)  34 (34.3)  9 (9.1)  2 |
| Histology Clear Cell  Non Clear Cell | 24 (80.0)  6 (20.0) | 51 (71.8)  20 (28.2) | 75 (74.3)  26 (25.7) |
| Performance status 0-1  >2 | 24 (80.0)  6 (20.0) | 49 (69.0)  22 (31.0) | 73 (72.3)  28 (27.7) |
| IMDC score prognosis Good  Intermediate  Poor  Missing | 3 (10.3)  19 (65.5)  7 (24.1)  1 | 10 (14.1)  44 (61.9)  17 (23.9)  0 | 13 (13.0)  63 (63.0)  24 (24.0)  1 |
| Line numbers 1  2  3  ≥4 | 1 (3.3)  5 (16.7)  11 (36.7)  13 (43.3) | 2 (2.8)  26 (36.6)  16 (22.5)  27 (38.0) | 3 (3.0)  31 (30.7)  27 (26.7)  40 (39.6) |
| Duration of exposure under cabozantinib(mean[min-max]) (months) | 13.7 [2.1-32.1] | 11.5 [1.0-28.8] | 12.1 [1.0-32.1] |
| Outcomes |  |  |  |
| Disease control rate | 30 (100.0) | 64 (90.1) | 94 (93.1) |
| Objective response rate | 16 (53.3) | 21 (29.6) | 37 (36.6) |
| Progression-free survival, median (months) | 10.6 [7.8-12.9] | 8.9 [7.1-10.1] | 9.2 [7.6-10.6] |
| Overall survival, median (months) | 21.1 [18.3-62.2] | 18.1 [15.5-22.8] | 19.8 [17.5-23.7] |
| Overall grade 3-4 toxicities | 3 (10.0) | 32 (45.1) | 35 (34.7) |

BMI: Body Mass Index; IMDC: International Metastatic RCC Database Consortium

**Supplementary Table 2**: Description of age and IMDC score criteria in METEOR, CABOSUN and current study

| **Characteristics** | **METEOR^1^** | **CABOSUN^2^** | | **Current study*** |
| --- | --- | --- | --- | --- |
| Median age in years [min-max] | 63.0 [32.0-86.0] | | 63.0 [40.0-82.0] | 59.2 [28.0-78.0] |
| IMDC score  Good (%)  Intermediate (%)  Poor (%)  Missing n | 45%  42%  12%  0 | | 0%  81%  19%  0 | 14.3%  62.6%  23.1%  1 |
| Number of lines  First line  At least 2 lines | 71%  29% | | 100%  0% | 34%  66% |

*: These results come from the weight loss analysis (n=92)

IMDC: International Metastatic Renal Cell Carcinoma Database Consortium

^1^: Choueiri TK, Escudier B, Powles T, Mainwaring PN, Rini BI, Donskov F, et al. Cabozantinib versus Everolimus in Advanced Renal-Cell Carcinoma. N Engl J Med. 2015 Nov 5;373(19):1814–23.

^2^: Choueiri TK, Halabi S, Sanford BL, Hahn O, Michaelson MD, Walsh MK, et al. Cabozantinib Versus Sunitinib As Initial Targeted Therapy for Patients With Metastatic Renal Cell Carcinoma of Poor or Intermediate Risk: The Alliance A031203 CABOSUN Trial. J Clin Oncol Off J Am Soc Clin Oncol. 2017 20;35(6):591–7.

**Supplementary Figure 1.** Spider splot of change from baseline of weight (A) and skeletal muscle (B) during cabozantinib treatment (patient level).

| A.  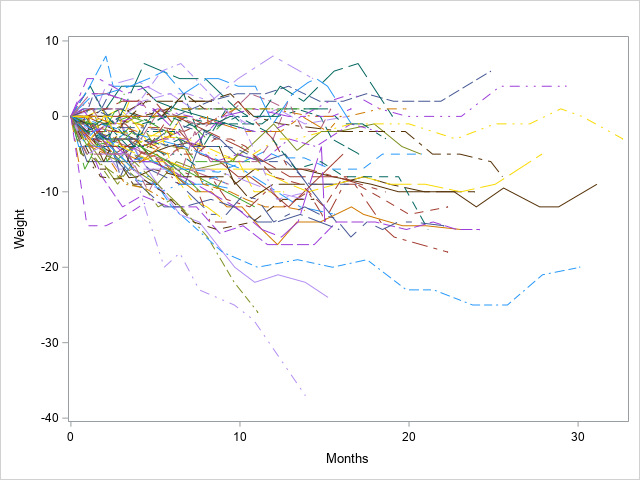 | B.  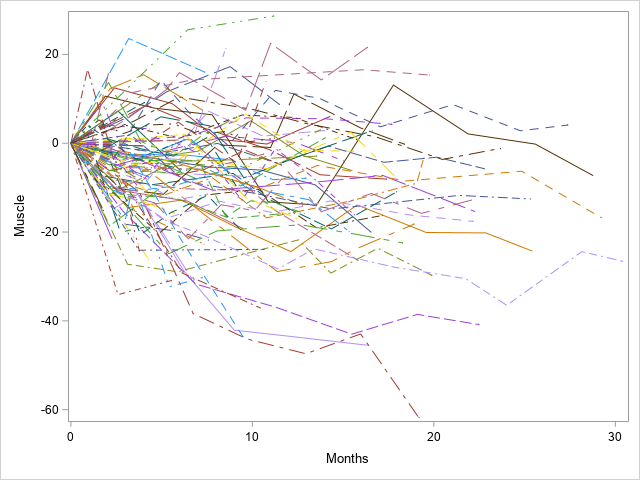 |
| --- | --- |

**Supplementary Figure 2.** Hypothesis of mechanism of WL during cabozantinib


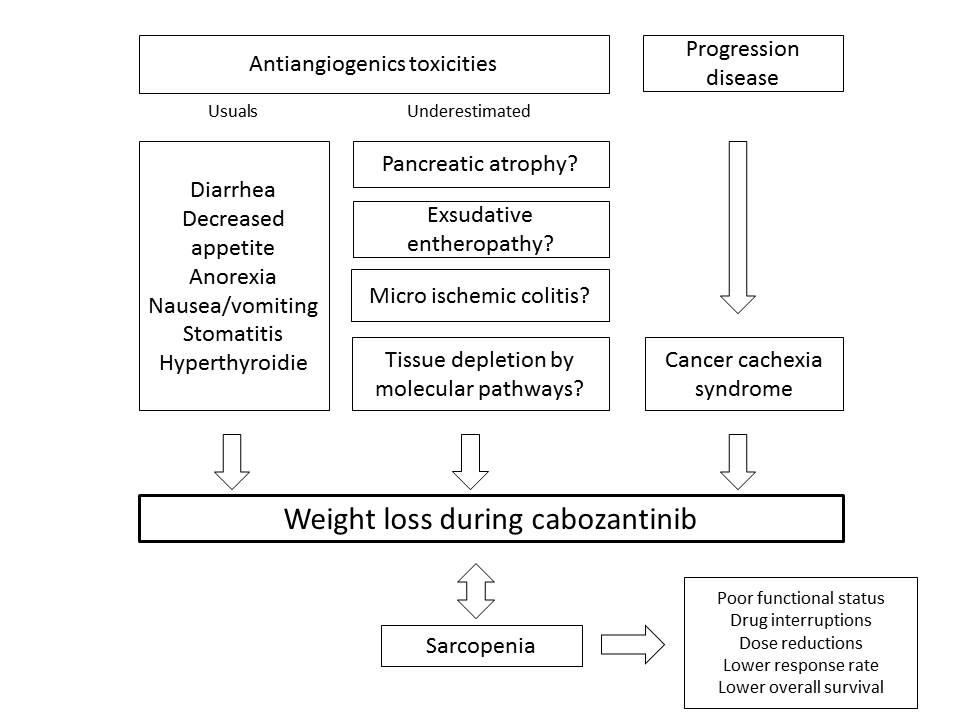

Supplement: Supplementary file 1 — Table S1: Patients'characteristics and outcomes according to sarcopenia status at cabozantininb baseline (n = 101) Table S2: Description of age and IMDC score criteria in METEOR, CABOSUN and current study Figure S1: Spider splot of change from baseline of weight (A) and skeletal muscle (B) during cabozantinib treatment (patient level) Figure S2: Hypothesis of mechanism of WL during cabozantinib [file JCSM-13-2405-s001.doc]
